# Detection and Classification of Cancer from Microscopic Biopsy Images Using Clinically Significant and Biologically Interpretable Features

**DOI:** 10.1155/2015/457906

**Published:** 2015-08-23

**Authors:** Rajesh Kumar, Rajeev Srivastava, Subodh Srivastava

**Affiliations:** Department of Computer Science and Engineering, Indian Institute of Technology (Banaras Hindu University), Varanasi 221005, India

## Abstract

A framework for automated detection and classification of cancer from microscopic biopsy images using clinically significant and biologically interpretable features is proposed and examined. The various stages involved in the proposed methodology include enhancement of microscopic images, segmentation of background cells, features extraction, and finally the classification. An appropriate and efficient method is employed in each of the design steps of the proposed framework after making a comparative analysis of commonly used method in each category. For highlighting the details of the tissue and structures, the contrast limited adaptive histogram equalization approach is used. For the segmentation of background cells, *k*-means segmentation algorithm is used because it performs better in comparison to other commonly used segmentation methods. In feature extraction phase, it is proposed to extract various biologically interpretable and clinically significant shapes as well as morphology based features from the segmented images. These include gray level texture features, color based features, color gray level texture features, Law's Texture Energy based features, Tamura's features, and wavelet features. Finally, the *K*-nearest neighborhood method is used for classification of images into normal and cancerous categories because it is performing better in comparison to other commonly used methods for this application. The performance of the proposed framework is evaluated using well-known parameters for four fundamental tissues (connective, epithelial, muscular, and nervous) of randomly selected 1000 microscopic biopsy images.

## 1. Introduction

Cancer detection has always been a major issue for the pathologists and medical practitioners for diagnosis and treatment planning. The manual identification of cancer from microscopic biopsy images is subjective in nature and may vary from expert to expert depending on their expertise and other factors which include lack of specific and accurate quantitative measures to classify the biopsy images as normal or cancerous one. The automated identification of cancerous cells from microscopic biopsy images helps in alleviating the abovementioned issues and provides better results if the biologically interpretable and clinically significant feature based approaches are used for the identification of disease.

About 32% of Indian population gets cancer at some point during their life time. Cancer is one of the common diseases in India which has responsibility to maximum mortality with about 0.3 million deaths per year [1]. The chances of getting affected by this disease are accelerated due to change in habits in the people such as increase in use of tobacco, deterioration of dietary habits, lack of activities, and many more. The possibility of cure from cancer is increased due to recent combined advancement in medicine and engineering. The chances of curing from cancer are primarily in its detection and diagnosis. The selection of the treatment of cancer totally depends on its level of malignancy. Medical professionals use several techniques for detection of cancer. These techniques may include various imaging modalities such as X-ray, Computer Tomography (CT) Scan, Positron Emission Tomography (PET), Ultrasound, and Magnetic Resonance Imaging (MRI) and pathological tests such as urine test and blood test.

For accurate detection of cancer pathologists use histopathology biopsy images, that is, the examination of microscopic tissue structure of the patient. Thus biopsy image analysis is a vital technique for cancer detection [2, 3]. Histopathology is the study of symptoms and indications of the disease using the microscopic biopsy images. To visualize various parts of the tissue under a microscope, the sections are dyed with one or more staining components. The main goal of staining is to reveal the components at cellular level and counterstains are used to provide color, visibility, and contrast. Hematoxylin-Eosin (H&E) is staining component that has been used by pathologists for over few decades. Hematoxylin stains cell nuclei which are blue in color while Eosin stains cytoplasm and connective tissues which are of pink color. The histology [4] is related to the study of cells in terms of structure, function, and interpretations of the tissue and cells. Microscopic biopsies are most commonly used for both disease screenings because of the less invasive natures. The characteristic of microscopic biopsy images has presence of isolated cells and cell clusters. The microscopic biopsy images are easier to analyze specimens compared to histopathology due to absence of noncomplicated structures [5]. The accurate manual identification of cancer from microscopic biopsy images has always been a major issue by the pathologists and medical practitioners observing cell or tissue structure under the microscope.

In histopathology, the cancer detection process normally consists of categorizing the image biopsy into cancerous one or noncancerous one [6]. In microscopic biopsy image analysis doctors and pathologists observe many of the abnormalities and categorize the sample based on various characteristics of the cell nuclei such as color, shape, size, and proportion to cytoplasm. High resolution microscopic biopsy provides reliable information for differentiating abnormal and normal tissues. The difference between normal and cancerous cells is shown in Table 1 [7].

For the detection and diagnosis of cancer from microscopic biopsy images, the histopathologists normally look at the specific features in the cells and tissue structures. The various common features used for the detection and diagnosis of cancer from the microscopic biopsy images include shape and size of cells, shape and size of cell nuclei, and distribution of the cells. The brief descriptions of these features are given as follows. 

*(A) Shape and Size of the Cells.* It has been observed that the overall shape and size of cells in the tissues are mostly normal. The cellular structures of the cancerous cells might be either larger or shorter than normal cells. The normal cells have even shapes and functionality. Cancer cells usually do not function in a useful way and their shapes are often not even. 

*(B) Size and Shape of the Cell's Nucleus.* The shape and size of the nucleus of a cancer cell are often not normal. The nucleus is decentralized in the cancer cells. The image of the cell looks like an omelet, in which the central yolk is the nucleus and the surrounding white is the cytoplasm. The nuclei of cancer cells are larger than the normal cells and deviated from the centre of the mass. The nucleus of cancer cell is darker. The segmentation step mainly focuses on separation of regions of interests (cells) from background tissues as well as separation of nuclei from cytoplasm. 

*(C) Distribution of the Cells in Tissue*. The function of each tissue depends on the distribution and arrangements of the normal cells. The numbers of healthy cells per unit area are less in the cancerous tissues. These adjectives of microscopic biopsy images have been included in shape and morphology based features, texture features, color based features, Color Gray Level Cooccurrence Matrix (GLCM), Law's Texture Energy (LTE), Tamura's features, and wavelet features which are more biologically interpretable and clinically significant.

The main aim of this paper is to design and develop a framework and a software tool for automated detection and classification of cancer from microscopic biopsy images using the abovementioned clinically significant and biologically interpretable features. This paper focuses on selecting an appropriate method for each design stage of the framework after making a comparative analysis of the various commonly used methods in each category. The various stages involved in the proposed methodology include enhancement of microscopic images, segmentation of background cells, features extraction, and finally the classification.

The rest of the paper has been structured as follows.  Section 2 describes the related works, Section 3 presents the methods and models, Section 4 describes the results and discussions, and finally Section 5 draws the conclusion of the work presented in this paper.

## 2. Related Works

In recent years, few works have been reported in the literature for the design and development of tools for automated cancer detection from microscopic biopsy images. Kumar and Srivastava [9] presented detailed reviews on the computer aided diagnosis (CAD) for cancer detection from microscopic biopsy images. Demir and Yener [10] also presented a method for automatic diagnosis of biopsy image. They presented a cellular level diagnosis system using image processing techniques. Bhattacharjee et al. [11] presented a review on computer aided diagnosis system to detect cancer from microscopic biopsy images using image processing techniques.

Bergmeir et al. [12] proposed a model to extract the texture features by using local histograms and GLCM. The quasisupervised learning algorithm operates on two datasets, the first one having normal tissues labeled only indirectly and the second one containing an unlabeled collection of mixed samples of both normal and cancer tissues. This method was applied on the dataset of 22,080 vectors with reduced dimensionality, 119 from 132. The regions having the cancerous tissues were accurately identified having true positive rate 88% and false positive rate 19%, respectively, by using manual ground truth dataset.

Mouelhi et al. [13] used Haralick's textures features [14], histogram of oriented gradients (HOG), and color component based statistical moments (CCSM) features selection and extraction approaches to classify the cancerous cells from microscopic biopsy images. The various features used in this paper are contrast, correlation, energy, homogeneity, GLCM texture features [14], RGB, gray level, and HSV.

Huang and Lai [15] presented a methodology for segmentation and classification techniques for histology images based on texture features and by using SVM the maximum classification accuracy obtained is 92.8%.

Landini et al. [16] presented a method for morphologic characterization of cell neighborhoods in neoplastic and preneoplastic tissue of microscopic biopsy images. In this paper, authors presented watershed transforms to compute the cell and nuclei area and other parameters. The distance measure of the neighborhood value has been used for calculating the neighborhood complexity with reference to the v-cells. The best classification which has been obtained by *K*NN classifier is 83% for dysplastic and neoplastic classes and 58% of correct classification.

Sinha and Ramkrishan [17] extracted some features of microscopic biopsy images which include eccentricity, area ratio, compactness, average values of color components, energy entropy, correlation, and area of cells and nucleus. The classification accuracy obtained by Bayesian, *K*-nearest neighbor, neural networks, and support vector machine was 82.3%, 70.60%, 94.1%, and 94.1%, respectively.

Kasmin et al. [18] extracted the features of microscopic biopsy images including area, perimeter, convex area, solidity, major axis length, orientation filled area, eccentricity, ratio of cell and nucleus area, circularity, and mean intensity of cytoplasm. The *K*NN and neural network classifier are used for classification accuracy 86% and 92%, respectively.

In this paper, a framework for automated detection and classification of cancer from microscopic biopsy images using clinically significant and biologically interpretable features is proposed and examined. For segmentation of images colour *k*-means based method is used. The various hybrid features which are extracted from the segmented images include shape and morphological features, GLCM texture features, Tamura features, Law's Texture Energy based features, histogram of oriented gradients, wavelet features, and color features. For classification purposes, *k*-nearest neighbor based method is proposed to be used. The efficacy of other classifiers such as SVM, random forest, and fuzzy *k*-means is also examined. For testing purposes, 2828 microscopic biopsy images available from histology database [8] are used. From the obtained results, it was observed that the proposed method is performing better in comparison to other methods discussed as above. The overall summary and comparison of the proposed method and other methods are presented in Table 6 in Section 4 of results and analysis.

## 3. Methods and Models

**Figure 1:**
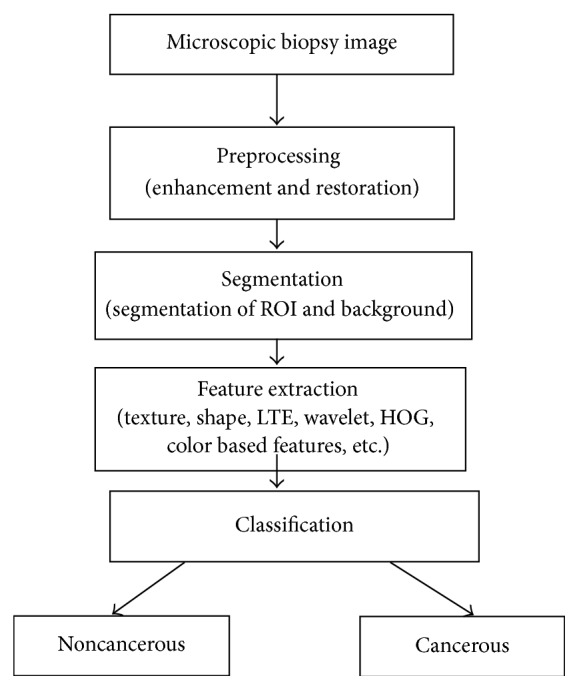
Model of automated cancer detection from microscopic biopsy images.

The detection and classification of cancer from microscopic biopsy images are a challenging task because an image usually contains many clusters and overlapping objects. The various stages involved in the proposed methodology include enhancement of microscopic images, segmentation of background cells, features extraction, and finally the classification. For the enhancement of the microscopic biopsy images, the contrast limited adaptive histogram equalization [19, 20] approach is used and for the segmentation of background cells *k*-means segmentation algorithm is used. In feature extraction phase, various biologically interpretable and clinically significant shape and morphology based features are extracted from the segmented images which include gray level texture features, color based features, color gray level texture features, Law's Texture Energy (LTE) based features, Tamura's features, and wavelet features. Finally, the *K*-nearest neighborhood (*K*NN), fuzzy *K*NN, and support vector machine (SVM) based classifiers are examined for classifying the normal and cancerous biopsy images. These approaches are tested on four fundamental tissues (connective, epithelial, muscular, and nervous) of randomly selected 1000 microscopic biopsy images. Finally, the performances of the classifiers are evaluated using well known parameters and from results and analysis, it is observed that the fuzzy *K*NN based classifier is performing better for the selected features set. The flowchart for the proposed work is given in Figure 1.

### 3.1. Enhancements

**Figure 2:**
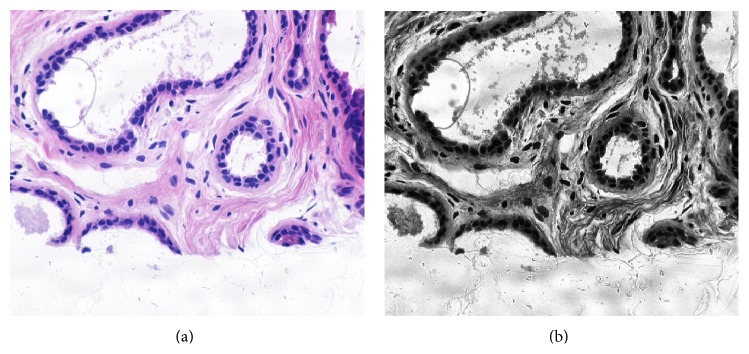
The original (a) and enhanced microscopic biopsy image with CLAHE (b).

The main purpose of the preprocessing is to remove a specific degradation such as noise reduction and contrast enhancement of region of interests. The biopsy images acquired from microscope may be defective and deficient in some respect such as poor contrast and uneven staining, and they need to be improved through process of image enhancement which increases the contrast between the foreground (objects of interest) and background [21]. The contrast limited adaptive histogram equalization (CLAHE) [20] approach is used for enhancement of microscopic biopsy images.  Figure 2 shows the original and enhanced image using contrast limited adaptive histogram equalization.

### 3.2. Segmentation

**Figure 3:**
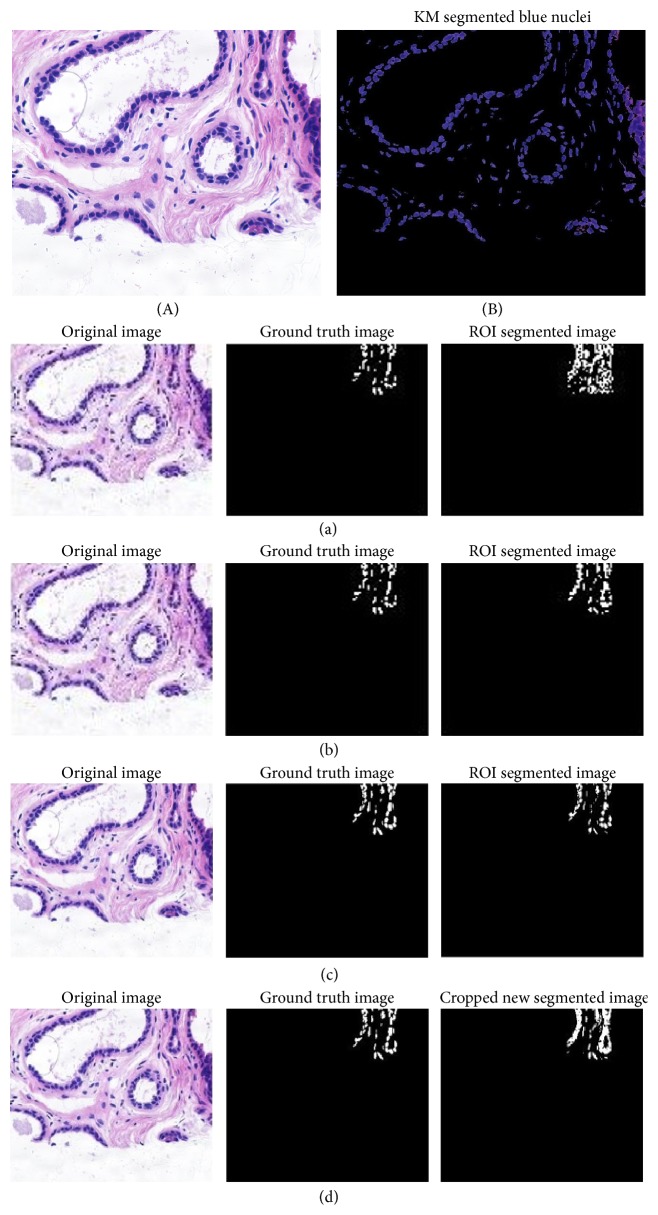
Original (A) and segmented microscopic biopsy image with *K*-means segmentation approach (B). (a) Original, ground truth, and ROI segmented by texture based segmentation. (b) Original, ground truth, and ROI segmented by FCM segmentation. (c) Original, ground truth, and ROI segmented by *k*-means segmentation. (d) Original, ground truth, and ROI segmented by color based segmentation.

Several segmentation methods have been adapted for cytoplasm, cell, and nuclei segmentation [22] from microscopic biopsy images like threshold based, region-based, and clustering based algorithms. However the selections of segmentation methods depend on the type of the features to be preserved and extracted. For the segmentation of ROI (region of interest), the ground truth (GT) of the images is manually cropped and created from histology dataset [8]. The *k*-means clustering based segmentation algorithms are used because of the preservation of the desired information. From the obtained results through experimentation it is observed that the clustering based algorithms specifically *k*-means based method are the best suited for microscopic biopsy images.  Figure 3 shows the original and *k*-means segmented microscopic biopsy image. For testing and experimentation purpose, twenty (20) microscopic biopsy images available from histology dataset [8] were used. These images were randomly selected for segmentation. The ground truth (GT) images are manually created by cropping the region of interest (ROI). The visual results of texture based segmentation, FCM segmentation, *K*-means segmentation, and color based segmentation [20, 23–26] are presented in Figures 3(a) to 3(d). Thus from the visual results obtained and reported in Figures 3(a) to 3(d), it is observed that the *k*-means clustering based segmentation method performs better in most of the cases as compared to other segmentation approaches under consideration for microscopic biopsy image segmentation.

Finally the ROI segmented image of microscopic biopsy is compared to ground truth images for the quantitative evaluation of various segmentation approaches for all 20 sample images from histology dataset. The performance of the various segmentation approaches such as *K*-means [27], fuzzy *c*-means [28], texture based segmentation [29], and color based segmentation [30] was evaluated in terms of various popular parameters of segmentation measures. These parameters include accuracy, sensitivity, specificity, false positive rate (FPR), probability random index (RI), global consistency error (GCE), and variance of information (VOI).

The brief description of few of these performance measures used in this paper is as follows.

*(i) Probability Random Index (PRI)*. Probability random index is the nonparametric measure of goodness of segmentation algorithms. Random index between test (*S*) and ground truth (*G*) is estimated by summing the number of pixel pairs with same label and number of pixel pairs having different labels in both *S* and *G* and then dividing it by total number of pixel pairs. Given a set of ground truth segmentations *G*_*k*_, the PRI is estimated using (1) such that *c*_*ij*_ is an event that describes a pixel pair (*i*, *j*) having same or different label in the test image *S*_test_

(1) PRIStest,Gk=1N/2∑∀i,j&i<jcijpij+1−cij1−pij.

*(ii) Variance of Information (VOI)*. The variation of information is a measure of the distance between two clusters (partitions of elements) [31]. Clustering with clusters is represented by a random variable *X*, *X* = {1,…, *k*} such that *P*_*i*_ = |*X*_*i*_ | /*n*, *i* ∈ *X*, and *n* = ∑_*i*_ *X*_*i*_ is the variation of information between two clusters *X* and *Y*.

Thus VOI(*X*, *Y*) is represented using

(2) $$\begin{array}{c} \text{VOI}\left(\;X,Y\;\right)=H\left(\;X\;\right)=H\left(\;Y\;\right)-2I\left(\;X,Y\;\right), \end{array}$$

where *H*(*X*) is entropy of *X* and *I*(*X*, *Y*) is mutual information between *X* and *Y*. VOI(*X*, *Y*) measures how much the cluster assignment for an item in clustering *X* reduces the uncertainty about the item's cluster in clustering *Y*.

*(iii) Global Consistency Error (GCE)*. The GCE is estimated as follows: suppose segments *s*_*i*_ and *g*_*j*_ contain a pixel, say *p*_*k*_, such that *s* ∈ *S*, *g* ∈ *G* where *S* denotes the set of segments that are generated by the segmentation algorithm being evaluated and *G* denotes the set of reference segments. To begin with, a measure of local refinement error is estimated using (3) and then it is used to compute local and global consistency errors, where *n* denotes the set of difference operation and *R*(*x*, *y*) represents the set of pixels corresponding to region *x* that includes pixel *y*. Using (3) [31] the global consistency error (GCE) is computed using (4) where *n* denotes the total number of pixels of the image. GCE quantify the amount of error in segmentation (0 signifies no error and 1 indicates no agreement):

(3) Esi,gj,pk=Rsi,pk∖Rgj,pkRsi,pk,

(4) GCES,G=1nmin⁡∑iES,G,pi,∑iES,G,pi.

**Figure 4:**
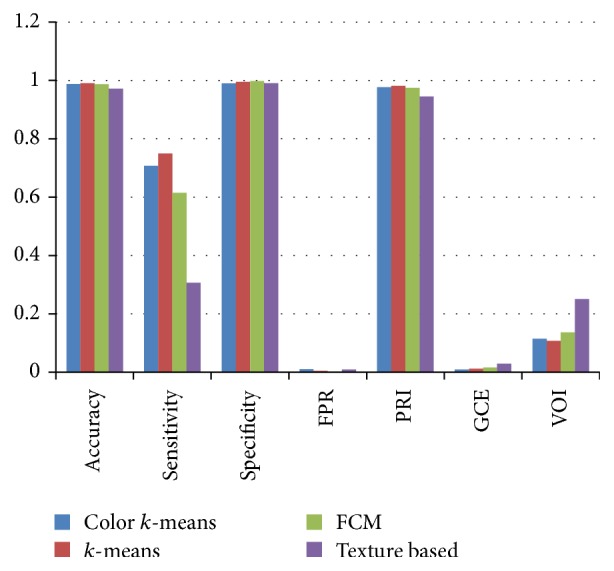
Comparisons of various segmentation methods on the basis of average accuracy, sensitivity, specificity, FPR, PRI, GCE, and VOI for 20 sample images from histology dataset [8].

Table 2 and Figure 4 show the comparison of various segmentation algorithms on the basis of average accuracy, sensitivity, specificity, FPR, PRI, GCE, and VOI for 20 sample images taken from histology dataset [8]. From Table 2 and Figure 4, it is observed that *k*-means, color *k*-means, fuzzy *c*-means, and texture based methods are performing better at par in terms of accuracy, specificity, and PRI segmentation measures but except for *k*-means based segmentation methods other methods are not performing better in terms of other important parameters. Only the *K*-means based segmentation algorithm is associated with larger value of accuracy, sensitivity, specificity, and random index (RI) and smaller value of FPR, GCE, and VOI in comparison to other methods and hence it is better in comparison to others. Hence, *k*-means based segmentation is the only method which performs better in terms of all parameters and that is why it is chosen as the segmentation method in the proposed framework for cancer detection from microscopic biopsy images.

### 3.3. Feature Extraction

After segmentation of image features are extracted from the regions of interest to detect and grade potential cancers. Feature extraction is one of the important steps in the analysis of biopsy images. The features are extracted at tissue level and cell level of microscopic biopsy images for better predictions. To better capture the shape information, we use both region-based and contour-based methods to extract anticircularity, area irregularity, and contour irregularity of nuclei as the three shape features to reflect the irregularity of nuclei in biopsy images. The cellular level feature focuses on quantifying the properties of individual cells without considering spatial dependency between them. In biopsy images for a single cell, the shape and morphological, textural, histogram of oriented gradients and wavelet features are extracted. The tissue level features quantify the distribution of the cells across the tissue; for that, it primarily makes use of either the spatial dependency of the cells or the gray level dependency of the pixels.

Based on these characteristics, some important shape and morphological based features are explained as follows.

*(i) Nucleus Area (A)*. The nucleus area can be represented by nucleus region containing total number of pixels; it is shown in

(5) $$\begin{array}{c} A=\overset{n}{\underset{i=1}{\sum }}\;\overset{m}{\underset{j=1}{\sum }}B\left(\;i,j\;\right), \end{array}$$

where *A* is nucleus area and *B* is segmented image of *i* rows and *j* columns. 

*(ii) Brightness of Nucleus*. The average value of intensity of the pixels belonging to the nucleus region is known as nucleus brightness. 

*(iii) Nucleus Longest Diameter (NLD)*. The largest circle's diameter circumscribing the nucleus region is known as nucleus longest diameter; it is shown in

(6) $$\begin{array}{c} \text{NLD}=\sqrt{{\left(x_{1}-x_{2}\right)}^{2}+{\left(y_{1}-y_{2}\right)}^{2}}, \end{array}$$

where *x*_1_, *y*_1_ and *x*_2_, *y*_2_ are end points on major axis. 

*(iv) Nucleus Shortest Diameter (NSD).* This is represented through smallest circle's diameter circumscribing the nucleus region. It is represented in

(7) $$\begin{array}{c} \text{NSD}=\sqrt{{\left(x_{2}-x_{1}\right)}^{2}+{\left(y_{2}-y_{1}\right)}^{2}}, \end{array}$$

where *x*_1_, *y*_1_ and *x*_2_, *y*_2_ are end points on minor axis. 

*(v) Nucleus Elongation.* This is represented by the ratio of the shortest diameter to the longest diameter of the nucleus region, shown in

(8) $$\begin{array}{c} \text{Nucleus elongation}=\frac{\text{NLD}}{Perimeter}. \end{array}$$

*(vi) Nucleus Perimeter (P)*. The length of the perimeter of the nucleus region is represented using

(9) $$\begin{array}{c} P=\text{Even count}+\sqrt{2}\left(\;\text{odd count}\;\right)\text{ unit.} \end{array}$$

*(vii) Nucleus Roundness (γ*). The ratio of the nucleus area to the area of the circle corresponding to the nucleus longest diameter is known as nucleus compactness, shown in

(10) $$\begin{array}{c} \gamma =\frac{A}{P}=\frac{4\pi \times \text{Area}}{P^{2}}. \end{array}$$

*(viii) Solidity*. Solidity is ratio of actual cell/nucleus area to convex hull area shown in

(11) $$\begin{array}{c} \text{Solidity}=\frac{Area}{Convex\;\;Area}. \end{array}$$

*(ix) Eccentricity*. The ratio of major axis length and minor axis length is known as eccentricity and defined in

(12) $$\begin{array}{c} \text{Eccentricity}=\frac{\text{Length of mejor Axis}}{\text{Length of minor Axis}}. \end{array}$$

*(x) Compactness*. Compactness is the ratio of area and square of the perimeter. It is formulated as

(13) $$\begin{array}{c} \text{Compactness}=\frac{\text{Area}}{{Perimeter}^{2}}. \end{array}$$

There are seven sets of features used for computing the feature vector of microscopic biopsy images explained as follows. 

*(i) Texture Features (F1–F22)*. [32–34] Autocorrelation, contrast, correlation, cluster prominence, cluster shade, difference variance, dissimilarity, energy, entropy, homogeneity, maximum probability, sum of squares, sum average, sum variance, sum entropy, difference entropy, information measure of correlation 1, information measure of correlation 2, inverse difference (INV), inverse difference normalized (INN), and inverse difference moment normalized are major texture features which can be calculated using equations of the texture features. 

*(ii) Morphology and Shape Feature (F23–F32)*. In papers [35, 36] authors describe the shape and morphology features. The considered shape and morphological features in this paper are area, perimeter, major axis length, minor axis length, equivalent diameter, orientation, convex area, filled area, solidity, and eccentricity. 

*(iii) Histogram of Oriented Gradient (HOG) (F33–F68)*. Histogram of oriented gradient is one of the good features set to deify the objects [32]. In our observation it will be included for better and accurate identification of objects present in microscopic biopsy images. 

*(iv) Wavelet Features (F69–100)*. Wavelets are small wave which is used to transform the signals for effective processing [3]. The wavelets are useful in multiresolution analysis of microscopic biopsy images because they are fast and give better compression as compared to other transforms. The Fourier transform converts a signal into a continuous series of sine waves, but the wavelet precedes it in both time and frequency. This accounts for the efficiency of wavelet transforms [37]. Daubechies wavelets have been used because they have fractal structures and they are useful in the case of microscopic biopsy images. In this paper mean, entropy, energy, contrast homogeneity, and sum of wavelet coefficients are taken into consideration. 

*(v) Color Features (F101–F106).* The components of these models are hue, saturation, and value (HSV) [34]. This is represented by the six sided pyramids, the vertical axis behaves as brightness, the horizontal distance from the axis represents the saturation, and the angle represents the hue. Here mean and standard deviation of HSV components are taken as features. 

*(vi) Tamura's Features (F107–F109).* Tamura's features are computed on the basis of three fundamental texture features: contrast, coarseness, and directionality [3]. Contrast is the measure of variety of the texture pattern. Therefore, the larger blocks that make up the image have a larger contrast. It is affected by the use of varying black and white intensities [32]. Coarseness is the measure of granularity of an image [32]; thus coarseness can be represented using average size of regions that have the same intensity [38]. Directionality is the measure of directions of the grey values within the image [32]. 

*(vii) Law's Texture Energy (LTE) (F110–F115)*. These features are texture description features which mainly used average gray level, edges, spots, ripples, and wave to generate vectors of the masks. Law's mask is represented by the features of an image without using frequency domain [39]. Laws significantly determined that several masks of appropriate sizes were very instructive for discriminating between different kinds of texture features present in the microscopic biopsy images. Thus its classified samples are based on expected values of variance-like square measures of these convolutions, called texture energy measures. The LTE mask method is based on texture energy transforms applied to the image classification used to estimate the energy within the pass region of filters [40].

Table 3 provides the distribution of name of the feature type and the number of features selected for the classification of microscopic biopsy images.

### 3.4. Classification

The classification of microscopic biopsy images is the most challenging task for automatic detection of cancer from microscopic biopsy images. Classification might provide the answer whether microscopic biopsy is benign or malignant. For classification purposes, many classifiers have been used. Some commonly used classification methods are artificial neural networks (ANN), Bayesian classification, *K*-nearest neighbor classifiers, support vector machine (SVM), and random forest (RF). Supervised machine learning approaches are used for the classification of microscopic biopsy images. There are various steps involved in the supervised learning approaches. First step is to prepare the data (feature set), the second step is to choose an appropriate algorithm, the third step is to fit a model, the fourth step is to train the fitted model, and then the final step is to use fitted model for prediction. The *K*-nearest neighborhood (*K*NN), fuzzy *K*NN and support vector machine (SVM), and random forest classifiers are used for classifying the normal and cancerous biopsy images.

## 4. Results and Discussions

The proposed methodologies were implemented with MATLAB 2013b, on dataset of digitized at 5x magnification on PC with 3.4 GHz Intel Core i7 processor, 2 GB RAM, and windows 7 platform.

For the testing and experimentation purposes, a total of 2828 histology images from the histology image dataset (histologyDS2828) and annotations are taken from a subset of images related to above database [8]. The image distributions based on the fundamental tissue structures in the histology dataset include Connective-484, Epithelial-804, Muscular-514, and Nervous-1026 microscopic biopsy images with magnifications 2.5x, 5x, 10x, 20x, and 40x. Although the method is magnification independent, in this work the results are provided on samples digitized at 5x magnification. The features extracted from microscopic biopsy images must be biologically interpretable and clinically significant for better diagnosis of cancer.  Table 4 provides the brief description of dataset used for identification of cancer from microscopic biopsy images.

The proposed methodology for detection and diagnosis of cancer detection from microscopic biopsy images consists of the stages of images enhancement, segmentation, feature extraction, and classification.

The contrast limited adaptive histogram equalization (CLAHE) is used for enhancement of microscopic biopsy images, because it has ability to better highlight the regions of interests in the images as tested through experimentation.

To better preserve the desired information in microscopic biopsy images during segmentation process, the various clustering and texture based segmentation approaches were examined. For microscopic biopsy images it is required to discover as much as possible the nuclei information in order to make reliable and accurate detection and diagnosis based on cells and nuclei parameters. From results and analysis presented in Section 4, *k*-means segmentation algorithm [40] was used for segmenting the microscopic biopsy images as it performs better in comparison to other methods. During segmentation process of *k*-means clustering method, the number of clusters *k* was set to *k* = 3. Further, to find the center of the clusters, squared Euclidean distance measures are used as similarity measures.

In feature extraction phase, various biologically interpretable and clinically significant shape and morphology based features were extracted from the segmented images which include gray level texture features (F1–F22), shape and morphology based features (F23–F32), histogram of oriented gradients (F33–F68), wavelet features (F69–F100), color based features (F101–F106), Tamura's features (F107–F119), and Law's Texture Energy (F110–F115) based features. Finally a 2D matrix of 2828 × 115 feature matrix was formed using all the feature sets, where 2828 are the number of microscopic images in the dataset and 115 are the total number of features extracted.

Randomly selected 1000 data/samples were used for testing various classification algorithms. The 10-fold cross validation approach was used to partition the data in training and testing sets. Thus 900 data/samples were used for training purposes and 100 data/samples were used for testing purposes. The *K*-nearest neighbor (*K*NN) is a simple classifier in which a feature vector is assigned. For *K*NN classification the numbers of nearest neighbor (*k*) were set to 5, and Euclidean distance matrix and the “nearest” rule to decide how to classify the sample were used. The proposed method was also tested by using support vector machine (SVM) based classifier for linear kernel function with 10-fold cross validation methods. In SVM classification model, the kernel's parameters and soft margin parameter *C* play vital role in classification process; the best combination of *C* and *γ* was selected by a grid search with exponentially growing sequences of *C* and *γ*. Each combination of parameter choices was checked using cross validations (10-fold), and the parameters with best cross validation accuracy were selected. For SVM's linear kernel function, quadratic programming (QP) optimization parameter was used to find separating hyperplane. In the case of random forest the value by default is 500 trees and mtry = 10.

The performance of classifiers was calculated using confusion matrix of size 2 × 2 matrix and the value of TP, TN, FP, and FN was calculated. The performance parameters accuracy, sensitivity, and specificity were calculated using (14)–(19).

The fundamental definitions of these performance measures could be illustrated as follows.

*Accuracy*. The classification accuracy of a technique depends upon the number of correctly classified samples (i.e., true negative and true positive) [40] and is calculated as follows:

(14) $$\begin{array}{c} Accuracy=\frac{TP+TN}{N}\times 100, \end{array}$$

where *N* is the total number of samples present in the microscopic biopsy images.

*Sensitivity*. Sensitivity is a measure of the proportion of positive samples which are correctly classified [41]. It can be calculated using

(15) $$\begin{array}{c} Sensitivity=\frac{TP}{TP+FN}, \end{array}$$

where the value of sensitivity ranges between 0 and 1, where 0 and 1, respectively, mean worst and best classification. 

*Specificity*. Specificity is a measure of the proportion of negative samples that are correctly classified [42]. The value of sensitivity is calculated using

(16) $$\begin{array}{c} Specificity=\frac{TN}{TN+FP}. \end{array}$$

Its value ranges between 0 and 1, where 0 and 1, respectively, mean worst and best classification. 

*Balanced Classification Rate (BCR)*. The geometric mean of sensitivity and specificity is considered as balance classification rate [43, 44]. It is represented by

(17) $$\begin{array}{c} \text{BCR}=\sqrt{Sensitivity\times Specificity}. \end{array}$$

*F-Measure*. *F*-measure is a harmonic mean of precision and recall. It is defined by using

(18) Precision=TPTP+FP,Recall=TPTP+FN,F-measure=2×Precision×RecallPrecision+Recall.

The value of *F*-measure ranges between 0 and 1, where 0 means the worst classification and 1 means the best classification.

*Matthews's Correlation Coefficient (MCC)*. MCC is a measure of the eminence of binary class classifications [43]. It can be calculated using the following formula:

(19) MCC=TP×TN−FP×FNTP+FNTP+FPTN+FNTN+FP.

Its value ranges between −1 and +1, where −1, +1, and 0, respectively, correspond to worst, best, at random prediction.

*Discussions of Results*.  Table 5 shows classification results of the proposed framework for four different tissues of microscopic biopsy images containing cancer and noncancer cases tested using four popular classifiers like *k*-nearest neighbor, SVM, fuzzy *K*NN, and random forest.

**Figure 5:**
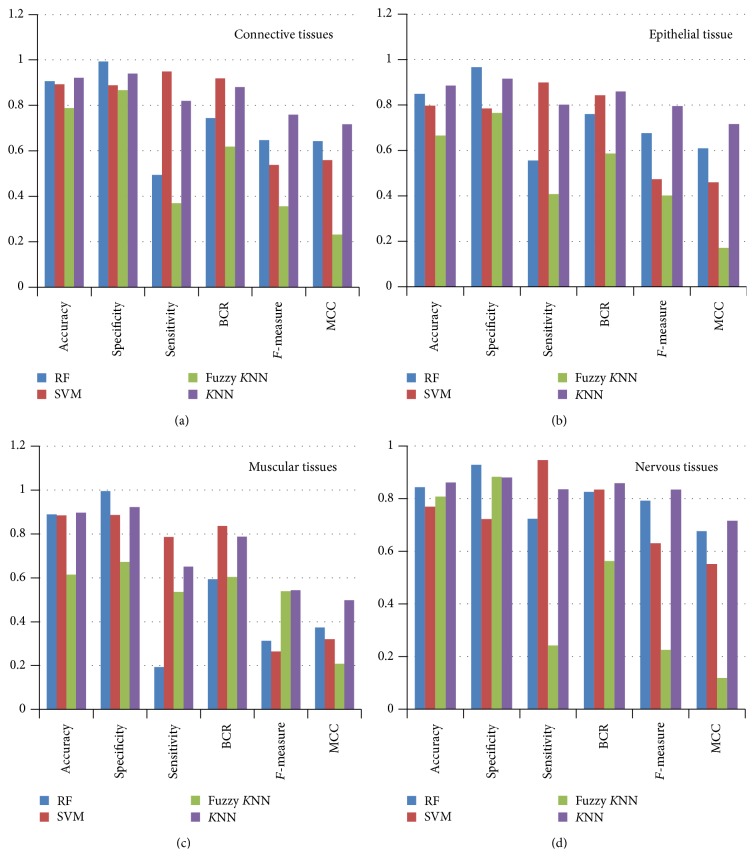
Performance analysis of classifiers with four fundamental tissues: connective tissue as (a), epithelial tissue as (b), muscular tissue as (c), and nervous tissue as (d).

From Table 5 and Figure 5(a) the following observations are made for sample test cases containing connective tissues.

(i) For the identification of cancer from biopsy images of connective tissues in the case of *K*NN, the average value of accuracy, specificity, sensitivity, BCR, *F*-measure, and MCC is 0.921909, 0.940164, 0.819922, 0.880263, 0.759395, and 0.717455, respectively.
(ii) For the identification of cancer from biopsy of connective tissues in the case of SVM, the average value of accuracy, specificity, sensitivity, BCR, *F*-measure, and MCC is 0.89245, 0.888438, 0.948297, 0.918756, 0.538314, and 0.55879, respectively.
(iii) For the identification of cancer from biopsy of connective tissues in the case of fuzzy *K*NN, the average value of accuracy, specificity, sensitivity, BCR, *F*-measure, and MCC is 0.787879, 0.867476, 0.370074, 0.618789, 0.356613, and 0.231013, respectively.
(iv) For the identification of cancer from biopsy of connective tissues, in the case of random forest classifier, the average value of accuracy, specificity, sensitivity, BCR, *F*-measure, and MCC is 0.907245, 0.993668, 0.493996, 0.743832, 0.647373, and 0.642137, respectively.

From Table 5 and Figure 5(b) the following observations are made for sample test cases containing epithelial tissues.

(i) For the identification of cancer from biopsy images of epithelial tissues in the case of *K*NN, the average value of accuracy, specificity, sensitivity, BCR, *F*-measure, and MCC is 0.884727, 0.916446, 0.801733, 0.859435, 0.795319, and 0.71626, respectively.
(ii) For the identification of cancer from biopsy of epithelial tissues in the case of SVM, the average value of accuracy, specificity, sensitivity, BCR, *F*-measure, and MCC is 0.796998, 0.7851, 0.898525, 0.842279, 0.472804, and 0.4587, respectively.
(iii) For the identification of cancer from biopsy of epithelial tissues in the case of fuzzy *K*NN, the average value of accuracy, specificity, sensitivity, BCR, *F*-measure, and MCC is 0.665834, 0.76465, 0.407057, 0.585984, 0.401181, and 0.17053, respectively.
(iv) For the identification of cancer from biopsy of epithelial tissues, in the case of random forest classifier, the average value of accuracy, specificity, sensitivity, BCR, *F*-measure, and MCC is 0.849306, 0.966243, 0.555332, 0.760788, 0.675868, and 0.609494, respectively.

From Table 5 and Figure 5(c) the following observations are made for sample test cases containing muscular tissues.

(i) For the identification of cancer from biopsy images of muscular tissues in the case of *K*NN, the average value of accuracy, specificity, sensitivity, BCR, *F*-measure, and MCC is 0.897321, 0.923277, 0.650761, 0.787092, 0.543009, and 0.49783, respectively.
(ii) For the identification of cancer from biopsy of muscular tissues in the case of SVM, the average value of accuracy, specificity, sensitivity, BCR, *F*-measure, and MCC is 0.884379, 0.886718, 0.786303, 0.83681, 0.263764, and 0.320547, respectively.
(iii) For the identification of cancer from biopsy of muscular tissues in the case of fuzzy *K*NN, the average value of accuracy, specificity, sensitivity, BCR, *F*-measure, and MCC is 0.614958, 0.672503, 0.535894, 0.604364, 0.538571, and 0.208941, respectively.
(iv) For the identification of cancer from biopsy of muscular tissues, in the case of random forest classifier, the accuracy, specificity, sensitivity, BCR, *F*-measure, and MCC are 0.889878, 0.995023, 0.193145, 0.594084, 0.313309, and 0.37318, respectively.

From Table 5 and Figure 5(d) the following observations are made for sample test cases containing nervous tissues.

(i) For the identification of cancer from biopsy images of nervous tissues in the case of *K*NN, the average value of accuracy, specificity, sensitivity, BCR, *F*-measure, and MCC is 0.861763, 0.880866, 0.835733, 0.858482, 0.834116, and 0.716492, respectively.
(ii) For the identification of cancer from biopsy of nervous tissues in the case of SVM, the average value of accuracy, specificity, sensitivity, BCR, *F*-measure, and MCC is 0.769545, 0.723056, 0.946068, 0.834923, 0.630126, and 0.552038, respectively.
(iii) For the identification of cancer from biopsy of nervous tissues in the case of fuzzy *K*NN, the accuracy, specificity, sensitivity, BCR, *F*-measure, and MCC are 0.808453, 0.882722, 0.242776, 0.562835, 0.225886, and 0.11837, respectively.
(iv) For the identification of cancer from biopsy of nervous tissues, in the case of random forest classifier, the average value of accuracy, specificity, sensitivity, BCR, *F*-measure, and MCC is 0.843102, 0.92827, 0.723262, 0.825766, 0.792403, and 0.676888, respectively.

From the above discussions for all four categories of test cases, it is observed that the *K*NN is performing better in comparison to other classifiers for the identification of cancer from biopsy images of nervous tissues.

From all above observations, it is concluded that the *K*NN classifier is producing better results in comparison to other methods for the case of biopsy images of connective tissues. The maximum values of the accuracy, sensitivity, and specificity are 0.9552, 0.9615, and 0.9543, respectively. The *k*-nearest neighbor classifier is also performing better for all cases as well as that was discussed above. Table 6 gives a comparative analysis of the proposed framework with other standard methods available in the literature. From Table 6, it can be observed that the proposed method is performing better in comparison to all other methods.

## 5. Conclusions

An automated detection and classification procedure was presented for detection of cancer from microscopic biopsy images using clinically significant and biologically interpretable set of features. The proposed analysis was based on tissues level microscopic observations of cell and nuclei for cancer detection and classification. For enhancement of microscopic biopsy images contrast limited adaptive histogram equalization based method was used. For segmentation of images *k*-means clustering method was used. After segmentation of images, a total of 115 hybrid sets of features were extracted for 2828 sample histology images taken from histology database [8]. After feature extraction, 1000 samples were selected randomly for classification purposes. Out of 1000 samples of 115 features, 900 samples were selected for training purposes and 100 samples were selected for testing purposes. The various classification approaches tested were *K*-nearest neighborhood (*K*NN), fuzzy *K*NN, support vector machine (SVM), and random forest based classifiers. From Table 5 we are in position to conclude that *K*NN is the best suited classification algorithm for detection of noncancerous and cancerous microscopic biopsy images containing all four fundamental tissues. SVM provides average results for all the tissues types but not better than *K*NN. Fuzzy *K*NN is comparatively a less good classifier. RF classifier provides very low sensitivity and down accuracy rate as compared to *K*NN classifier for this dataset. Hence, from experimental results, it was observed that *K*NN classifier is performing better for all categories of test cases present in the selected test data. These categories of test data are connective tissues, epithelial tissues, muscular tissues, and nervous tissues. Among all categories of test cases, further it was observed that the proposed method is performing better for connective tissues type sample test cases. The performance measures for connective tissues dataset in terms of the average accuracy, specificity, sensitivity, BCR, *F*-measure, and MCC are 0.921909, 0.940164, 0.819922, 0.880263, 0.759395, and 0.717455, respectively.

## Figures and Tables

**Table 1:** Difference between normal and cancerous cells [7].

Normal cells	Cancerous cells	Description of cancerous cells
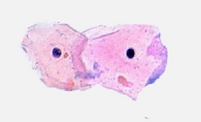	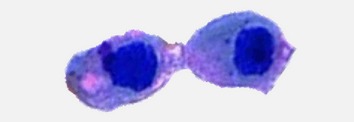	Large and variably shaped nuclei

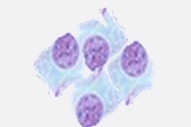	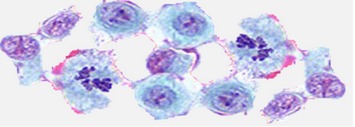	Many dividing cells and disorganized arrangements

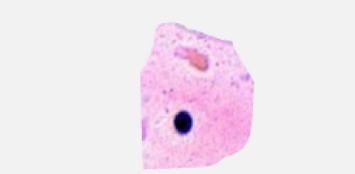	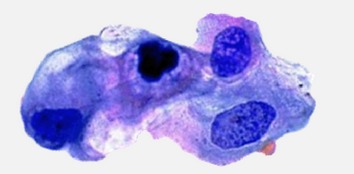	Variation in size and shape of nuclei

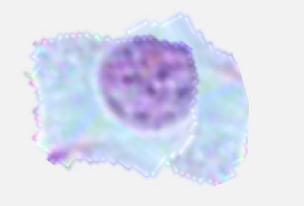	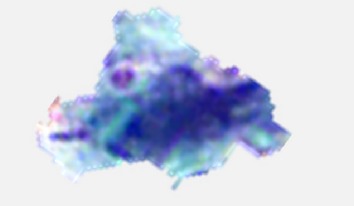	Loss of normal feature (shape and morphology)

**Table 2:** Quantitative evaluation of segmentation methods on the basis of average values of various performance metrics for a set of 20 microscopic images [8].

	Accuracy	Sensitivity	Specificity	FPR	PRI	GCE	VOI
Color *k*-means	0.987799	0.707025	0.989218	0.010782	0.975985	0.009205	0.115479
*k*-means	**0.990444**	**0.748991**	**0.994933**	**0.005067**	**0.981119**	**0.012839**	**0.10818**
FCM	0.987008	0.614717	0.998235	0.001765	0.974447	0.015902	0.136348
Texture based	0.97144	0.306398	0.990445	0.009555	0.944609	0.029276	0.250797

**Table 3:** The distribution of various features extracted from images and their ranges.

Name of features	Number of features (range F1–F115)
Texture features	22 (F1–F22)
Morphology and shape feature	10 (F23–F32)
Histogram of oriented gradient (HOG)	36 (F33–F68)
Wavelet features	32 (F69–100)
Color features	6 (F101–F106)
Tamura's features	3 (F107–F109)
Law's Texture Energy	16 (F110–F115)

**Table 4:** Image distribution of fundamental tissues dataset of 2828 histology images [8].

Fundamental tissue	Number of images
Connective	484
Epithelial	804
Muscular	514
Nervous	1026
**Total**	**2828**

**Table 5:** Comparative performances of various classifiers for the chosen features for various tissue types.

	Accuracy	Specificity	Sensitivity	BCR	*F*-measure	MCC	Accuracy	Specificity	Sensitivity	BCR	*F*-measure	MCC
	Connective tissues						Epithelial tissues					
RF	0.907245	0.993668	0.493996	0.743832	0.647373	0.642137	0.849306	0.966243	0.555332	0.760788	0.675868	0.609494
SVM	0.89245	0.888438	0.948297	0.918756	0.538314	0.55879	0.796998	0.7851	0.898525	0.842279	0.472804	0.4587
FYZZY *K*NN	0.787879	0.867476	0.370074	0.618789	0.356613	0.231013	0.665834	0.76465	0.407057	0.585984	0.401181	0.17053
*K*NN	**0.921909**	**0.940164**	**0.819922**	**0.880263**	**0.759395**	**0.717455**	**0.884727**	**0.916446**	**0.801733**	**0.859435**	**0.795319**	**0.71626**

Muscular tissues							Nervous tissues					
RF	0.889878	0.995023	0.193145	0.594084	0.313309	0.37318	0.843102	0.92827	0.723262	0.825766	0.792403	0.676888
SVM	0.884379	0.886718	0.786303	0.83681	0.263764	0.320547	0.769545	0.723056	0.946068	0.834923	0.630126	0.552038
FUZZY *K*NN	0.614958	0.672503	0.535894	0.604364	0.538571	0.208941	0.808453	0.882722	0.242776	0.562835	0.225886	0.11837
*K*NN	**0.897321**	**0.923277**	**0.650761**	**0.787092**	**0.543009**	**0.49783**	**0.861763**	**0.880866**	**0.835733**	**0.858482**	**0.834116**	**0.716492**

**Table 6:** The comparison of the proposed method with other standard methods.

Authors (year)	Feature set used	Methods of classification	Parameters used (%)	Dataset used
Huang and Lai (2010) [15]	Texture features	Support vector machine (SVM)	Accuracy = 92.8	1000 × 1000, 4000 × 3000, and 275 × 275 HCC biopsy images

Di Cataldo et al. (2010) [45]	Texture and morphology	Support vector machine (SVM)	Accuracy = 91.77	Digitized histology lung cancer IHC tissue images

He et al. (2008) [46]	Shape, morphology, and texture	Artificial neural network (ANN) and SVM	Accuracy = 90.00	Digitized histology images

Mookiah et al. (2011) [47]	Texture and morphology	Error backpropagation neural network (BPNN)	Accuracy = 96.43, sensitivity = 92.31, and specificity = 82	83 normal and 29 OSF images

Krishnan et al. (2011) [48]	HOG, LBP, and LTE	LDA	Accuracy = 82	Normal-83 OSFWD-29

Krishnan et al. (2011) [48]	HOG, LBP, and LTE	Support vector machine (SVM)	Accuracy = 88.38	Histology images Normal-90 OSFWD-42 OSFD-26

Caicedo, et al. (2009) [8]	Bag of features	Support vector machine (SVM)	Sensitivity = 92 Specificity = 88	2828 histology images

Sinha and Ramkrishan (2003) [17]	Texture and statistical features	*K*NN	Accuracy = 70.6	Blood cells histology images

**The proposed approach **	**Texture, shape and morphology, HOG, wavelet color, Tamura's feature, and LTE**	**KNN**	**Average**: **accuracy** = **92.19**, **sensitivity** = **94.01**, **specificity** = **81.99**, **BCR** = **88.02**, **F-measure** = **75.94**, **MCC** = **71.74**	**2828 histology images **
